# Yeast culture dietary supplementation modulates gut microbiota, growth and biochemical parameters of grass carp

**DOI:** 10.1111/1751-7915.13261

**Published:** 2018-03-26

**Authors:** Han Liu, Juntao Li, Xianwu Guo, Yunxiang Liang, Weimin Wang

**Affiliations:** ^1^ College of Fisheries Key Lab of Freshwater Animal Breeding, Ministry of Agriculture Key Lab of Agricultural Animal Genetics, Breeding and Reproduction of Ministry of Education Huazhong Agricultural University 430070 Wuhan China; ^2^ Laboratorio de Biotecnología Genómica Centro de Biotecnología Genómica Instituto Politécnico Nacional Boulevard del Maestro esquina Elías Piña, Colonia Narciso Mendoza 88710 Ciudad Reynosa Tamaulipas Mexico; ^3^ Collaborative Innovation Center for Efficient and Health Production of Fisheries in Hunan Province 41500 Changde China; ^4^ Present address: College of Fisheries Huazhong Agricultural University Wuhan 430070 China

## Abstract

Gut microbiota contributes positively to the physiology of their host. Some feed additives have been suggested to improve livestock health and stimulate growth performance by modulating gut bacteria species. Here, we fed grass carp with 0 (control), 8% (Treat1), 10% (Treat2), 12% (Treat3) and 16% (Treat4) of yeast culture (YC) for 10 weeks. The gut microbiota was analysed by 16S rRNA gene V3‐4 region via an Illumina MiSeq platform. PCoA test showed that gut bacterial communities in the control and Treat3 formed distinctly separate clusters. Although all the groups shared a large size of OTUs as a core microbiota community, a strong distinction existed at genus level. Treat3 contained the highest proportion of the beneficial bacteria and obviously enhanced the capacity of amino acid, lipid metabolism and digestive system. In addition, Treat3 significantly improved the fish growth and increased the liver and serum T‐SOD activities while dramatically decreased the liver GPT and GOT. Collectively, these findings demonstrate the beneficial effects of YC feeding on gut microbiota, growth and biochemical parameters and Treat3 might be the optimal supplementation amount for grass carp, which opens up the possibility that a new feed additive can be developed for healthy aquaculture.

## Introduction

Aquaculture has emerged as one of the most promising and fastest growing industries, and healthy aquaculture technology and management is a major concern in aquaculture to provide high‐quality products for human consumption. Grass carp, *Ctenopharyngodon idellus* is one of the most importantly economic aquaculture species. It represents the largest freshwater aquaculture product and has great commercial value in the world. According to the latest statistics of Food and Agriculture Organization (FAO), global production of cultured or farmed grass carp is approximately 5.5 million tons in 2014, which account for 7.5% of global freshwater aquaculture production of this year (FAO, 2016).

In the past, antibiotics were used to prevent the spread of disease in commercial aquaculture. However, overuse of antibiotics in aquaculture led to an increasing concern over spread of antibiotic‐resistant bacterial genes in the environment and suppression of the aquatic animal's immune system (Cabello, 2006; Baquero *et al*., 2008). The restriction for use of antibiotics in aquaculture may increase fish disease rates and influence the total production of the world aquaculture. Therefore, along with the increasing demand for aquatic product, it is imperative to find some effective alternatives to improve the fish health and maintain efficiency of aquatic production. Recently, to replace antibiotics in aquaculture, some probiotics, prebiotics and other feed additives, as novel dietary supplements have increased a great deal of attention to improve fish health and growth (Nayak, 2010; Giri *et al*., 2013; Huang *et al*., 2015).

It is commonly known that vertebrate gut microbiome provide a number of benefits to their host health including improvement of growth performance, nutrient digestion, immune function and protection from invasive pathogens (Viaud *et al*., 2013; Blanton *et al*., 2016; Stanley *et al*., 2016). Accumulating evidence suggested that probiotics and prebiotics products could modulate the gut microbiota of both human beings and animals, with consequences for improvement of their physiology and health (Li *et al*., 2014; Johnson *et al*., 2015; Pourabedin *et al*., 2015). In particular, the prebiotics products, such as galactooligosaccharides, fructooligosaccharides (FOS), mannan‐oligosaccharides (MOS) and andxylo‐oligosaccharides (XOS), were widely used to modulate the gut microbiota diversity, selectively stimulate the growth of more beneficial bacteria in human (Tuohy *et al*., 2005), livestock (Li *et al*., 2014; De Maesschalck *et al*., 2015; Pourabedin *et al*., 2015) and fish species (Dimitroglou *et al*., 2010; Carda‐Diéguez *et al*., 2014; Guerreiro *et al*., 2016), and inhibit colonization of pathogenic bacteria by producing antimicrobial substances. For example, treatments with certain XOS modified the relative abundance of chicken microbial genera and increased the probiotic bacteria such as *Bifidobacteria* (Pourabedin *et al*., 2015). Dimitroglou *et al*. (2010) demonstrated that dietary MOS on gilthead sea bream affected the intestinal microbial species richness and diversity (Dimitroglou *et al*., 2010).

Recently, the yeast culture (YC), as one of the promising feed additives candidates with many benefits, has also been used to modulate the animal gut microbiota. For instance, dietary YC supplementation at 5 g kg^−1^ had a positive effect on growth performance of nursery pigs by modulating gut immune response (Shen *et al*., 2009). In human beings, dried yeast modulates both the luminal and mucosal gut microbiota and protects against inflammation (Possemiers *et al*., 2013). In addition, consistent supplement with yeast cell wall prebiotics significantly increases the proportion of Proteobacteria phyla and *Faecalibacterium* genus in chicken (Park *et al*., 2016). Similarly, yeast supplementation also exhibited a sensitive response of the hindgut microbial ecosystem in horses (Grimm *et al*., 2016). In fish species, the feed intake and weight gain were promoted by dietary supplementation of yeast extract in Nile tilapia and a more efficient defence response to disease was shown (Berto *et al*., 2016). Essa *et al*. (2011) evaluated the effects of different additive levels of yeast on the Egyptian African catfish (*Clarias gariepinus*), which indicated that adding high level of yeast recorded higher final body weight and growth rate. Although yeast supplementation could promote growth performances and immune response in several fish species, whether the YC could improve the structure and composition of gut microbiota and generate the beneficial bacteria in cyprinid grass carp is not clear.

In the present study, we used high‐throughput sequencing of the V3‐V4 region of 16S rRNA gene to assess the effects of the different YC dietary supplementation on the gut microbiota composition, diversity and metabolic capacity in grass carp. In addition, the growth performance and biochemical parameters were also determined. The main objective of this work was to clarify how the YC dietary treatment influences the fish gut microbiota by evaluating the variability among different YC dietary treatments and confirm which additive amount is optimal for grass carp.

## Results

### The influence of YC treatment on gut microbial diversity

**Figure 1:**
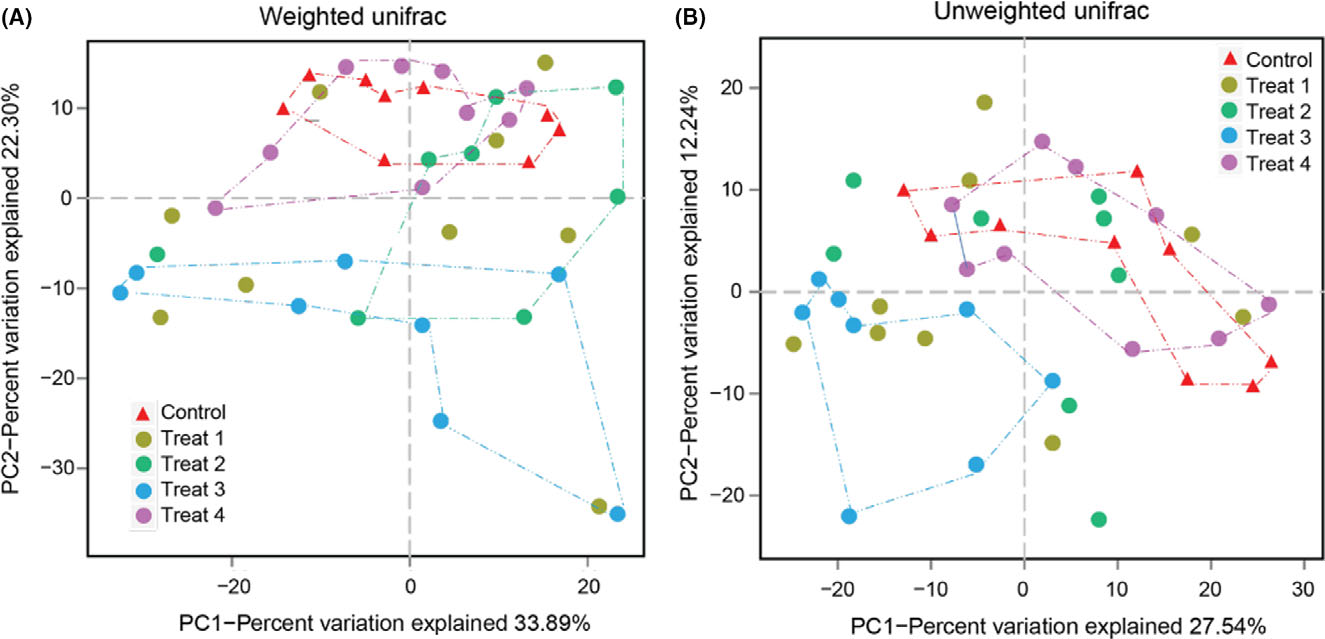
YC dietary treatments differently shift the gut microbial community structure of grass carp. Principle coordinates analyses (PCoA) of weighted (A) and unweighted (B) UniFrac distances show that gut communities cluster by different YC dietary treatments.

In total, 1.5 million quality‐controlled reads were generated from 16S rRNA gene V3 + V4 amplicons with an average of 34 774 reads per subject (ranging from 26 203 to 43 032) (Table S1). The assembled sequences had an average length of 437 bp. Operational taxonomic units (OTUs) clustering at 97% cutoff yielded a total of 6916 OTUs for the entire data set. The microbial complexity within and between samples was estimated on the basis of alpha‐diversity and beta‐diversity respectively. Tukey's HSD test for multiple comparisons found that there were no significant differences (*P *>* *0.05) in terms of alpha‐diversity based on the observed richness (OTUs, ACE, Chao1) and Shannon's or Simpson's diversity indices between the control and YC‐treated groups (Table 1). From the rarefaction curves, we found similar trend in the microbial diversity among the 43 specimens, approaching the saturation plateau (Fig. S1). In terms of beta‐diversity analysis based on the overall community composition revealed that the extent of similarity between gut microbiota clustered according to YC dietary treatments (Fig. 1). Our UniFrac principal coordinate analysis (PCoA) of 6,916 OTUs (grouped at 97% sequence identity) indicated a clear separation between the control and Treat3 group using both weighted (Fig. 1A) and unweighted (Fig. 1B) analysis. However, within the Treat1 and Treat2, samples of the same treatment have higher dispersion and did not show separate clustering to the control. It should also be noted that distinct clusters were observed in Treat4, but obviously clustered together to the control.

**Table 1:** Diversity of the gut microbiome was evaluated using OTUs defined at 97% sequence similarity

Groups	YC%	Sequenced library No.	Total filtered quality sequences	Richness estimates			Diversity estimates	
				OTUs	ACE	Chao1	Shannon	Simpson
Control	0	9	295510	148 ± 9.63	172 ± 8.44	171 ± 10.76	2.16 ± 0.084	0.23 ± 0.015
Treat1	8	9	314307	153 ± 6.89	174 ± 8.46	179 ± 9.67	2.47 ± 0.15	0.20 ± 0.032
Treat2	10	8	256851	166 ± 9.34	193 ± 9.78	197 ± 10.11	2.46 ± 0.55	0.21 ± 0.052
Treat3	12	8	316645	158 ± 4.65	180 ± 5.96	183 ± 4.91	2.66 ± 0.16	0.18 ± 0.037
Treat4	16	9	311973	179 ± 7.59	200 ± 9.33	199 ± 8.12	2.66 ± 0.21	0.17 ± 0.030

### YC treatments affect the taxonomic composition of gut microbiome

**Figure 2:**
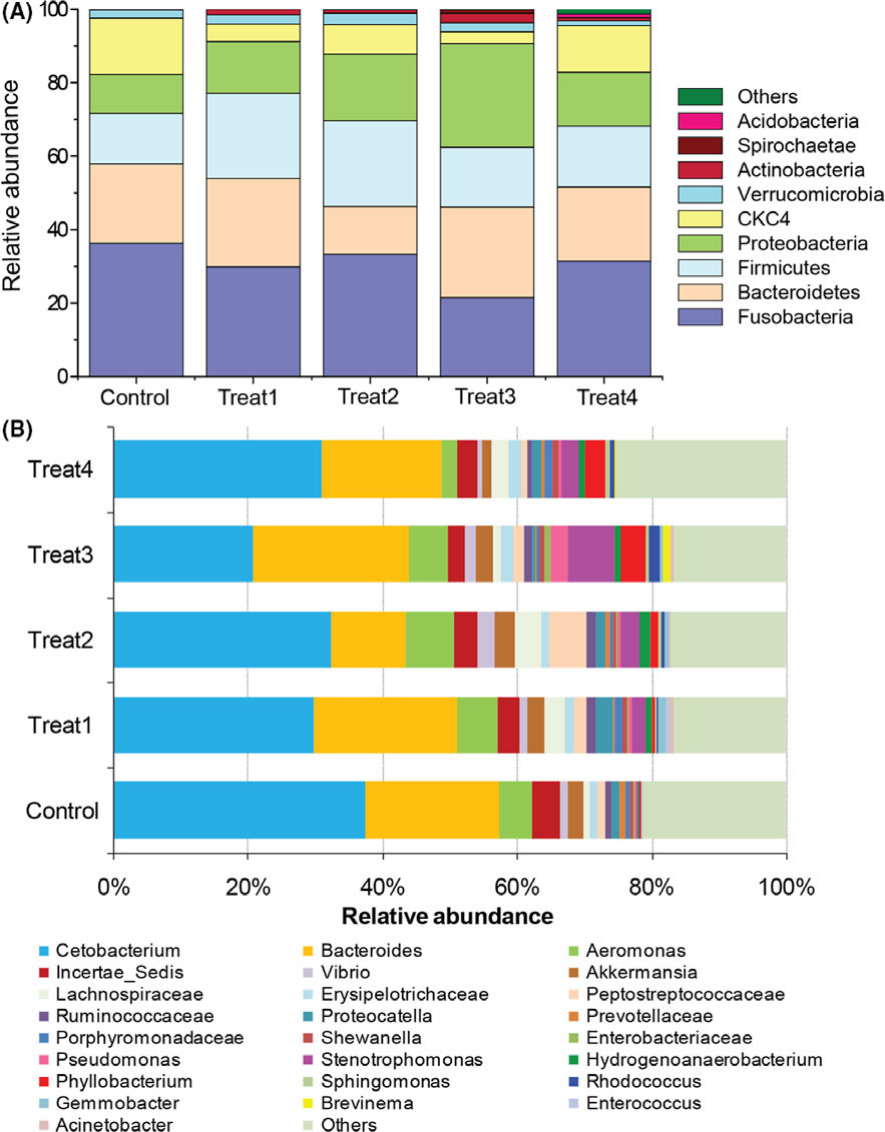
Taxonomic compositions of gut bacterial communities with different YC dietary treatments in grass carp. (A) The relative abundance of bacterial phyla. (B) Their relative abundance of each bacterial taxon (top 25 taxa) within a group at genus level. Control, *n* = 9; Treat1, *n* = 9; Treat2, *n* = 8; Treat3, *n* = 8; Treat4, *n* = 9.

In order to determine how the YC dietary treatments affect gut bacterial communities of grass carp, and how much of the YC supplement is optimal, the gut microbiomes of five groups with different YC treatments were compared at different taxonomic scales. Figure S2 illustrated approximately 99% of the total bacteria abundance was classified and the most abundant taxa (top 12) of bacteria were showed in all fish gut samples. Fusobacteria, Bacteroidetes, Firmicutes and Proteobacteria constituted the four most dominant phyla of bacterial communities in grass carp with different YC treatments, followed by CKC4 and Verrucomicrobia (Fig. 2A). Metastats‐based analysis of differential abundances among these phyla found that Fusobacteria (21.47 ±3.11%, *P *=* *0.050) and CKC4 (3.19 ± 0.71%, *P *=* *0.016) were significantly decreased in Treat3, whereas the Proteobacteria (27.80 ± 5.51%, *P *=* *0.048) and Actinobacteria (2.37 ± 0.45%, *P *=* *0.001) distinctively increased as compared with the control (Fig. S3). There is no significant difference of above‐mentioned bacterium in other YC treatments except CKC4 decreasing in Treat1 and Actinobacteria increasing in Treat1 and Treat2 (Fig. S3).

Inspection of taxonomic profiles at genus level for all samples, the most abundant taxa (top 25) of bacteria were shown in Fig. 2B, while the rest of the less frequent taxa were categorized as ‘others’. Three prominent members *Cetobacterium*,* Bacteroides* and *Aeromonas* were detected (Fig. 2B). Interestingly, the most distinct changes at the genus level included a considerable reduction of *Cetobacterium* (20.60 ± 4.04%, *P *=* *0.050) in Treat3 compared with the control and accompanied by a prominent increase of *Stenotrophomonas* (6.51 ± 1.83%, *P *=* *0.038), *Pseudomonas* (2.67 ± 0.91%, *P *=* *0.010), *Phyllobacterium* (3.61 ± 1.02%, *P *=* *0.027) and *Rhodococcus* (1.45 ± 0.62%, *P *=* *0.008) (Fig. S4). In contrast, no distinct differences in the relative abundance of the above‐mentioned bacteria were found in Treat1, Treat2 and Treat4 compared with the control, except the *Rhodococcus* in Treat2.

**Figure 3:**
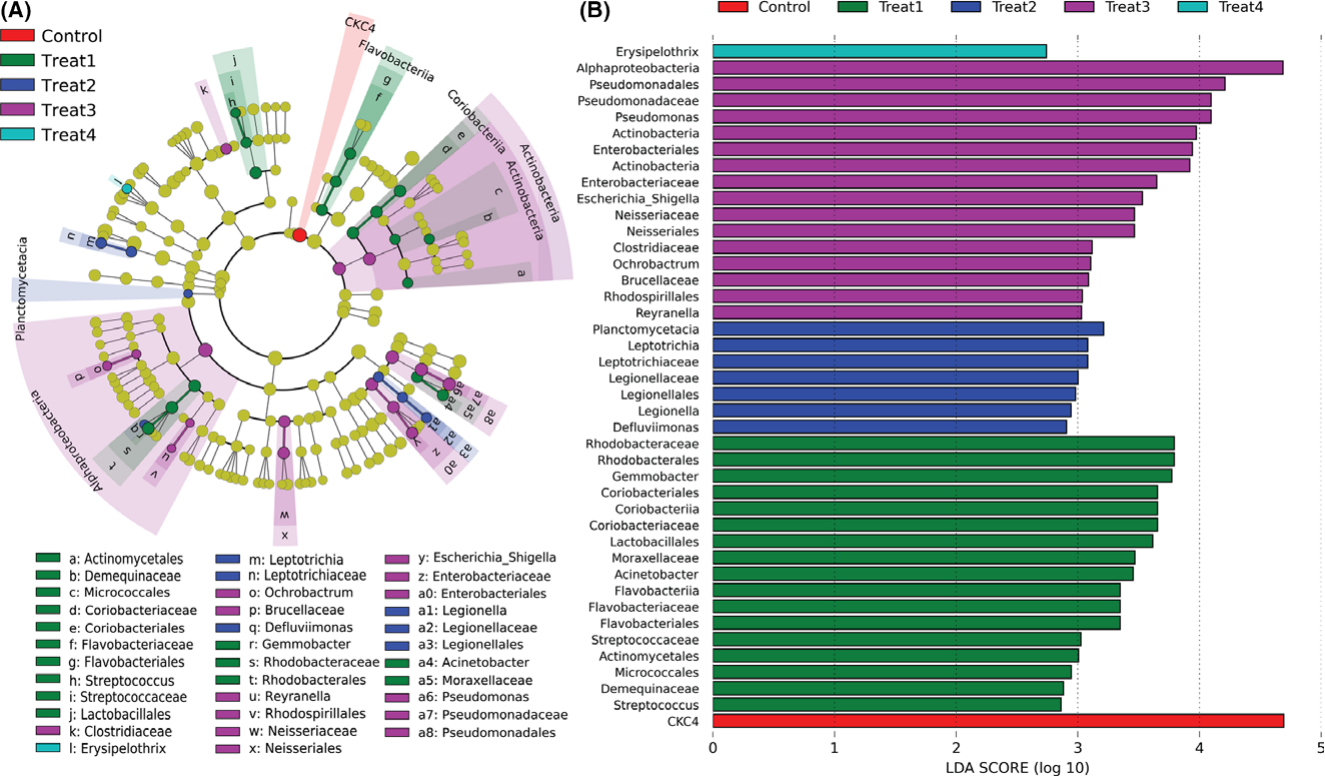
Differences in the gut microbiota of grass carp among the different YC dietary treatments. (A) Circular cladogram reporting LEfSe results presenting the identified OTUs distributed according to phylogenetic characteristics around the circle. Biomarker taxa are highlighted by coloured and shaded circles. Each circle's diameter is relative to abundance of taxa in community. (B) Linear discriminant analysis (LDA) effect size (LEfSe) results show that bacteria were significantly different in abundance between control and YC‐treated groups.

To further discriminate as many taxa as possible for meaningful comparisons, we also performed LEfSe to detect differential abundance of bacterial taxa among different YC treatments. As shown in Fig. 3A, the phylogenetic composition of gut microbiota was noticeably different among YC‐treated samples. The results showed that a total of 42 bacterial biomarkers at five different taxonomic levels were differentially abundant among the five groups (Fig. 3B). In comparison, Treat1 and Treat3 account for the majority of the 42 bacterial clades. Lactobacillales, Flavobacteriales and Rhodobacterales were the dominant orders in the Treat1 while Legionellales were mostly in the control. It is necessary to note that some important cellulose‐degrading bacteria including *Streptococcus*,* Leptotrichia* and *Pseudomonas* were differentially enriched in different YC treatments (Fig. 3B). Bacteria with differential abundance between control and Treat3 were also detected using LefSe (Fig. S5A). For example, the most differentially over represented taxa (LDA score > 3.0) in Treat3 were the genera *Rhodococcus*,* Chryseobacterium*,* Brevundimonas*,* Bradyrhizobium*,* Phyllobacterium*,* Serratia*,* Acinetobacter* and *Pseudomonas*, while in the control only the genera *Epulopiscium* and *Erysipelothrix* were the most differentially abundant taxa (Fig. S5B).

### Shared and unique microbial populations

**Figure 4:**
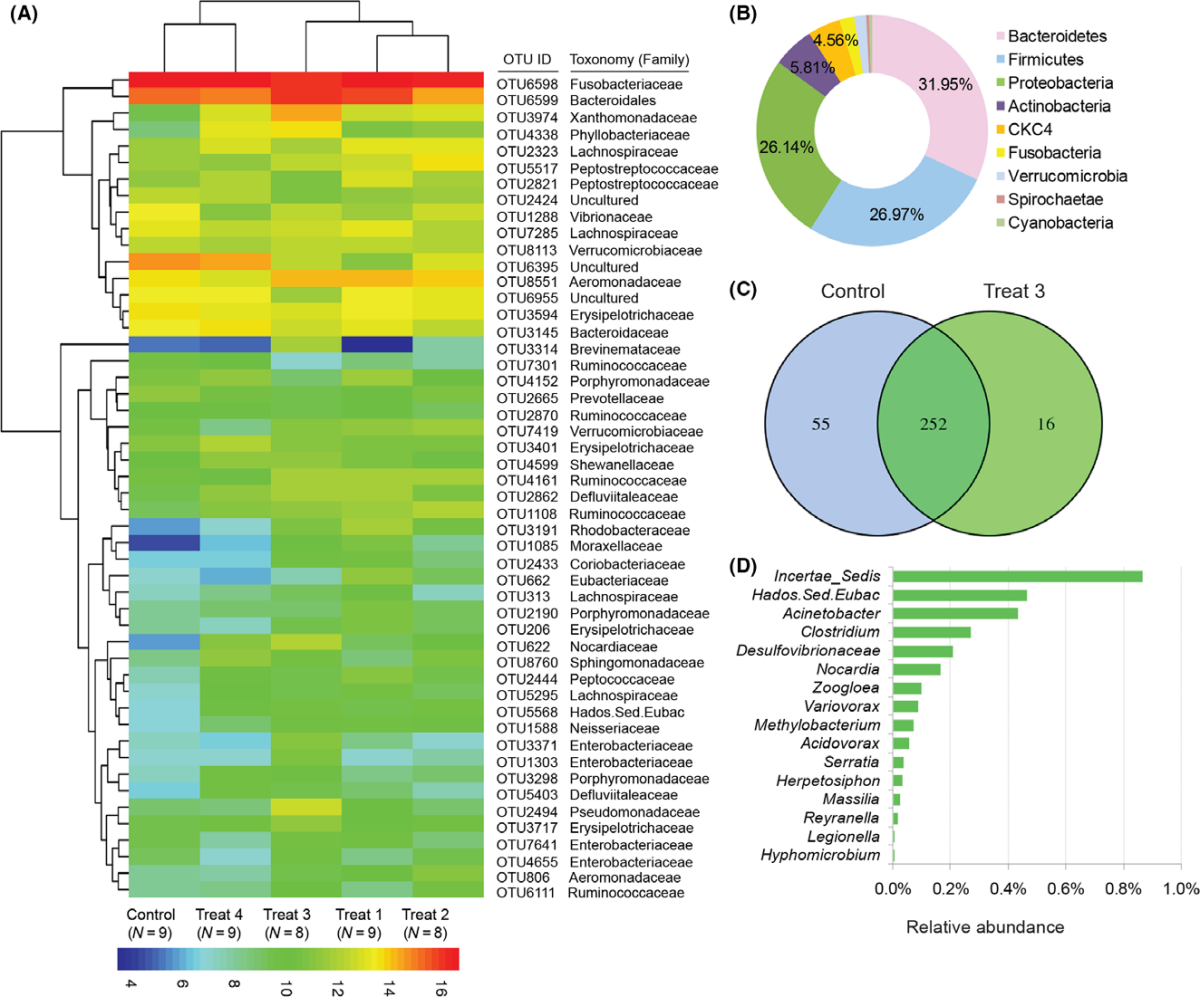
Shared and unique gut bacterial community composition of grass carp with different YC dietary treatments. (A) A heat map of changes in the relative abundances of the 50 most abundant OTUs summarized at the family level in gut microbial communities collected from YC dietary‐treated grass carp gut. (B) The composition of shared gut microbiota among five groups at the phylum level. (C) Number of shared OTUs between the Control and Treat3 and unique taxa. (D) The relative abundance of gut microbiota uniquely present in Treat3 at genus level.

A heatmap showed the abundance with large variation of the 50 most abundant bacterial taxa at family level among five groups (Fig. 4A). Interestingly, among these groups, the pattern of bacterial abundance in Treat4 was much similar to the control, which indicated that the high YC supplement might be with minor effect on gut microbiota compared with other groups. To further investigate the microbial community in different YC dietary treatments, the shared and unique OTUs were analysed through a Venn diagram. We identified 241 taxa present in all samples as the shared OTUs (Fig. S6). The most abundant shared OTUs at the phylum level were Bacteroidetes, Firmicutes, Proteobacteria, followed by Actinobacteria and CKC4 (Fig. 4B). Treat3 showed the largest difference compare to the control. Therefore, the unique and shared OTUs in control and Treat3 were analysed (Fig. 4C) and the five most abundant genera of the unique OTUs in Treat3 were *Incertae_Sedis*,* Acinetobacter*,* Clostridium*, Desulfovibrionaceae and *Nocardia* (Fig. 4D).

### Comparison of the potential beneficial bacteria abundance

**Figure 5:**
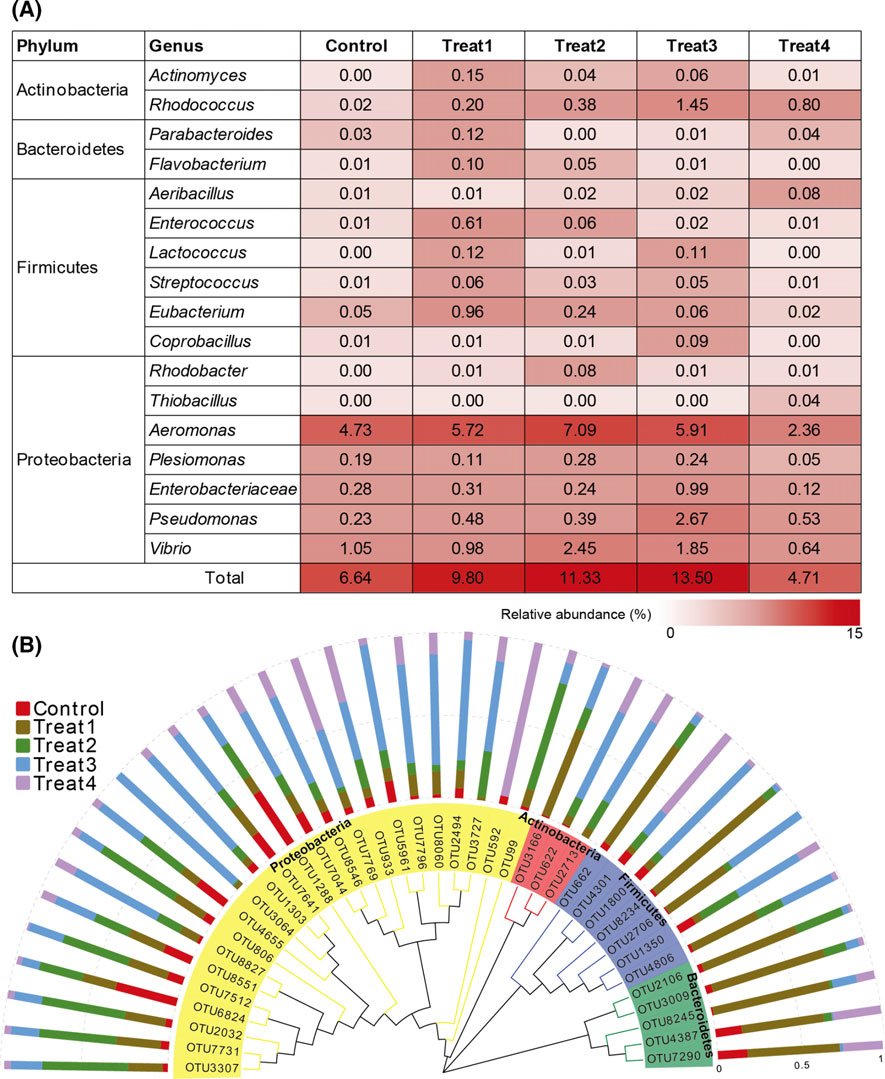
Relative abundance (%) and phylogenetic relationship of potential beneficial bacteria in gut samples from different YC dietary treatments. (A) A heat map of changes in the relative abundance of putative beneficial candidates in five trials. (B) Dendrogram of potential beneficial bacteria represented OTUs and their host occurrence patterns. Semicircle indicates the phylogenetic relationship of 17 above‐mentioned putative favourable species. Bars show the proportion of fish samples from different YC treatments in which the given OTUs is present.

In our present study, distinct and diverse putative beneficial candidates and taxonomic groups were identified in different dietary YC‐treated groups. Interestingly, the diversity of potential beneficial bacteria was relatively high, and a number of OTUs also had a high sequence similarity to these bacteria (Fig. 5). The relative abundance of these bacteria in Treat3 (13.50%) is twofold higher than that in the control, and close to Treat2 (11.33%), while a relatively low proportion was found in Treat4 (4.71%) (Fig. 5A). Among these favourable bacteria, the three most abundant were *Aeromonas*,* Vibrio* and *Pseudomonas* in all fish samples, but more in Treat2 and Treat3. To better visualize the OTUs diversity of the beneficial bacteria with a broader evolutionary context in different YC‐treated groups, a maximum likelihood phylogeny was constructed. As shown in Fig. 5B, a total of 39 OTUs were identified as 17 different favourable bacteria species. Among these OTUs, 24 were classified as Proteobacteria, seven as Firmicutes, five as Bacterioidetes and three as Actinobacteria, which differently distributed in the five groups.

### Predicted the functions of gut microbiota

**Figure 6:**
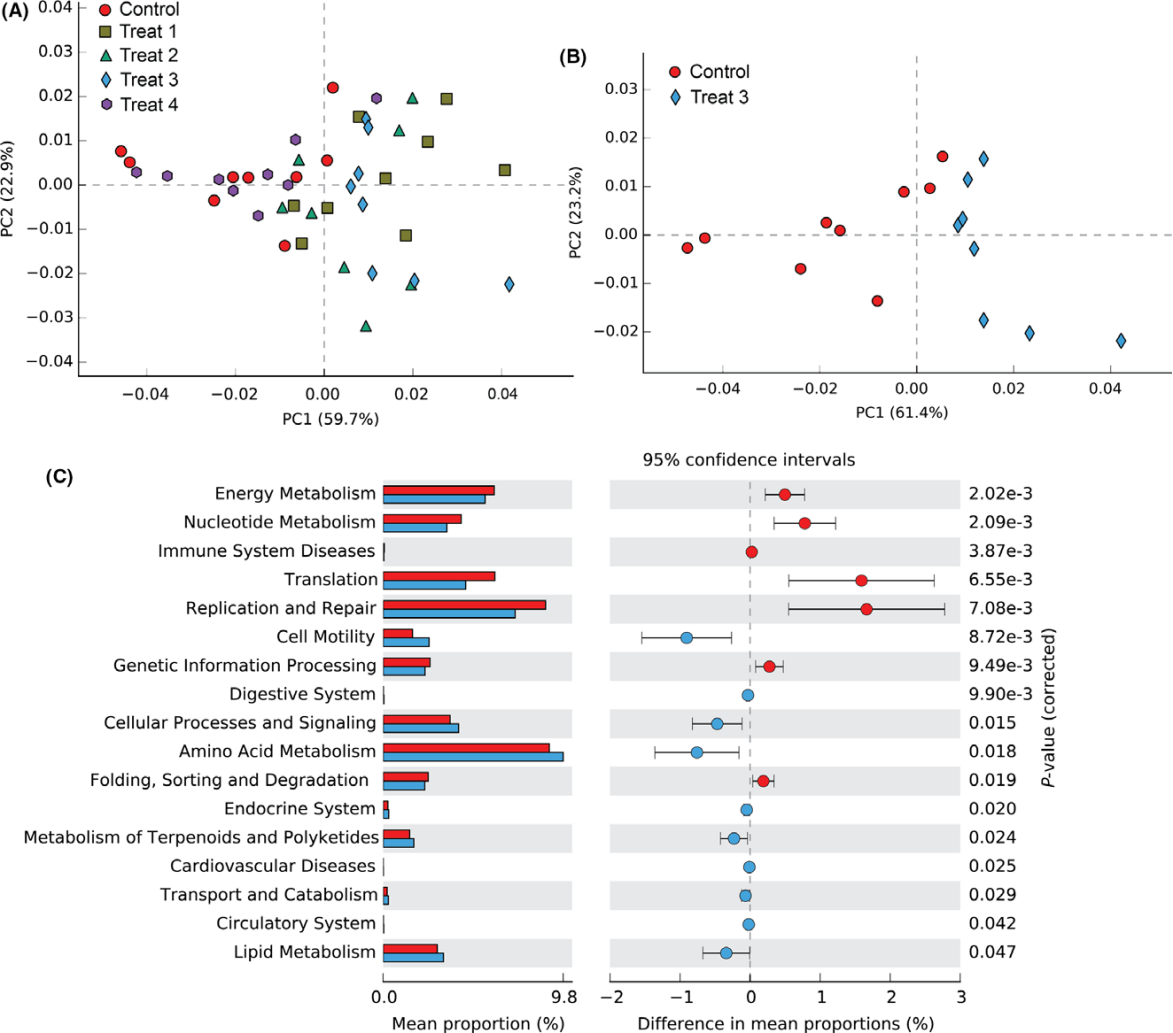
The predicted functional metagenomes of gut microbiota in grass carp with different YC dietary treatments. (A) Principal component analysis (PCA) predicted the functional metagenomes among control and YC dietary treatments. (B) PCA plot compared the taxonomic functional profiles between control and Treat3. (C) Extended error bar plot showing the normalized relative abundance of KEGG metabolic pathways (at level II) differing significantly between Control (red) and Treat3 (blue).

Based on the functionality prediction, a distinct difference in the KEGG Orthologues (KO) composition among different YC‐treated groups was detected (Fig. 6A), especially between the control and Treat1, Treat3 (Fig. 6B), respectively. In contrast, no obvious difference was detected between the control and Treat4 and they closely clustered each other. Pathways involving cell processes and signalling, digestive system, energy metabolism and immune system diseases were found over represented in each group (Fig. S7). Deeper analysis of the KEGG between control and Treat3 revealed that 17 of the 234 categories at levels II were shown to achieve a statistical significant difference at *P *<* *0.05 (Fig. 6C). Notably, significant elevation in cell motility, cellular processes and signalling, digestive system, amino acid metabolism, metabolism of terpenoids and polyketides and lipid metabolism pathways was observed in Treat3 whereas pathways related to the energy metabolism, nucleotide metabolism, translation, replication and repair, genetic information processing and folding, sorting and degradation were more detected in control.

### YC treatments improve the growth performances of grass carp

After 10 weeks of different concentrations of YC feeding, the YC dietary‐treated groups showed good growth performance as indicated by feed conversion ratio (FCR), condition factor (CF), gain rate (GR) and survival rate (SR) (Table 2). Although there were no significant differences between trials in SR and CF, significant differences in GR and FCR were detected (*P *<* *0.05). It is worth noting that Treat3 (12% YC) was with the highest GR. For the whole trial period (day 0–70), the FCR was significantly (*P *<* *0.05) more favourable for grass carp fed YC‐supplemented diet compared with the fish fed control diet, especially the Treat3 and Treat4. These results together with the increased GR show a biologically improved performance for fish fed YC.

**Table 2:** Growth performances of grass carp with yeast culture dietary treatments for 10 weeks

	Control	Treat1	Treat2	Treat3	Treat4
FCR	1.95 ± 0.24^a^	1.65 ± 0.05^ab^	1.71 ± 0.10^ab^	1. 60 ± 0.06^b^	1.56 ± 0.02^b^
CF	1.80 ± 0.02	1.80 ± 0.04	1.91 ± 0.05	1.77 ± 0.03	1.84 ± 0.05
GR	1.39 ± 0.05^a^	1.65 ± 0.35^b^	1.65 ± 0.01^b^	1.80 ± 0.03^c^	1.70 ± 0.02^bc^
SR (%)	95.61 ± 2.26	96.72 ± 1.93	94.43 ± 2.54	97.70 ± 1.18	91.15 ± 5.93

All data represent means ± SEM. In the same row, means with different letters are significantly different (*P *<* *0.05), means with the same letters are not significantly different (*P *>* *0.05). Absence of letters indicates no significant difference between treatments.

FCR, Feed conversion ratio; CF, Condition factor; GR, Gain rate; SR, Survival rate.

### Effects of YC treatments on liver and serum biochemical parameters

**Figure 7:**
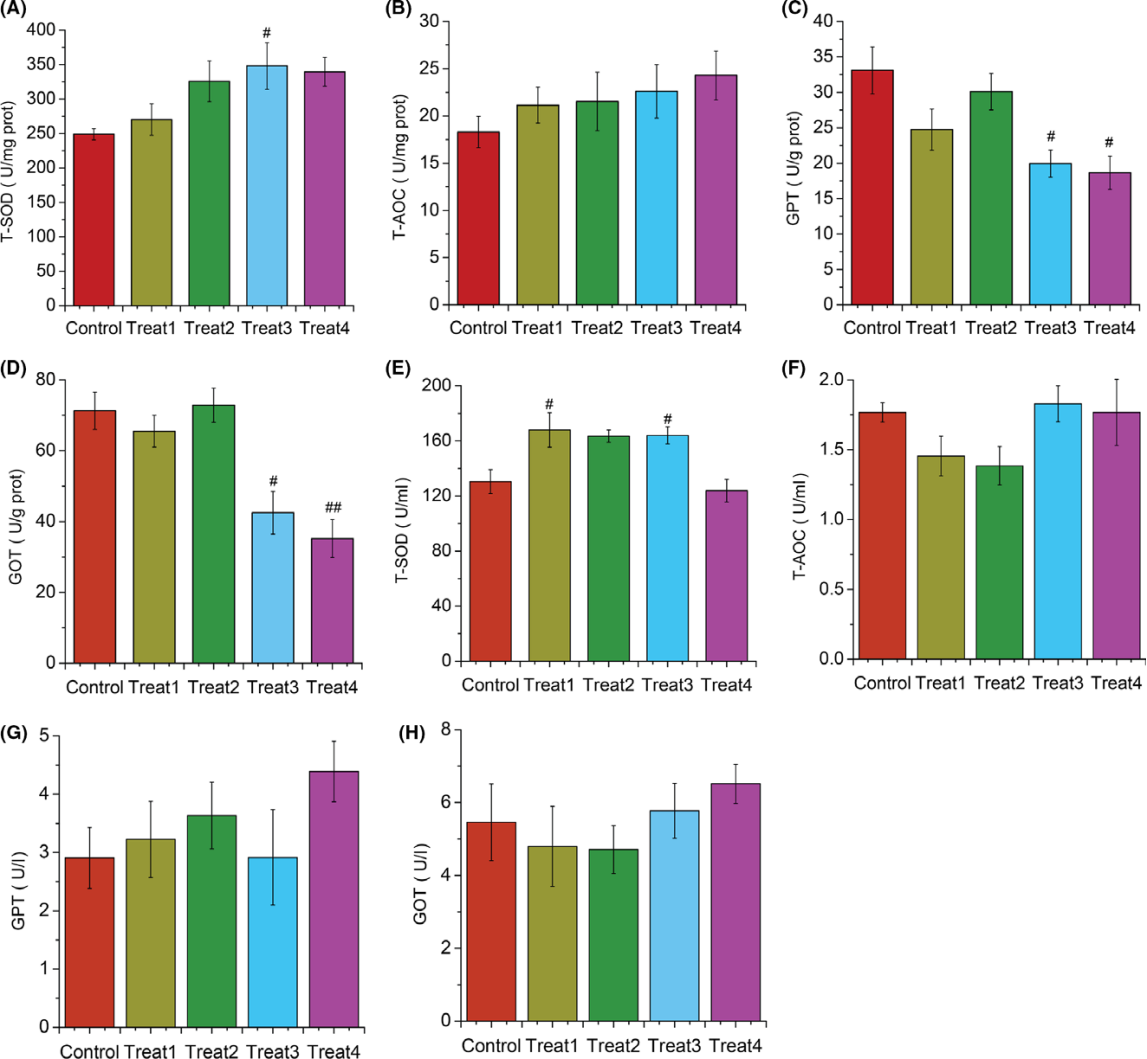
Effects of YC dietary treatment for 10 weeks on biochemical parameters in liver and serum of grass carp. (A) Liver T‐SOD. (B) Liver T‐AOC. (C) Liver GPT. (D) Liver GOT. (E) Serum T‐SOD. (F) Serum T‐AOC. (G) Serum GPT. (H) Serum GOT. Values are expressed as means ± S.E.M (*n* = 9). #*P *<* *0.05, ##*P *<* *0.01 compared with control group.

Despite the differences in the composition of gut microbiota and growth performance, the liver and serum T‐SOD, T‐AOC, GPT and GOT enzymes activities were differently influenced by YC dietary treatments in grass carp (Fig. 7) after 10 weeks of feeding. Specifically, 12% YC dietary treatment (Treat3) significantly increased (*P *<* *0.05) T‐SOD enzyme activity in the liver (Fig. 7A) and serum (Fig. 7E) while there was no difference (*P *>* *0.05) in T‐AOC enzyme activity in all YC‐treated groups (Fig. 7B and F). The liver GPT (Fig. 7C) and GOT (Fig. 7D) enzyme activities were dramatically decreased (*P *<* *0.05) in Treat3 and Treat4 (16% YC), as compared with the control, while the serum GPT (Fig. 7G) and GOT (Fig. 7H) among the groups with YC dietary treatments were similar.

## Discussion

More recently, high‐throughput sequencing of 16S rRNA gene amplicons has been used to explore the diversity and composition of gut microbiota in fish species, including three‐spined stickleback and Eurasian perch (Bolnick *et al*., 2014), fathead minnows (Narrowe *et al*., 2015), surgeonfishes (Miyake *et al*., 2015), Trinidadian guppies (Sullam *et al*., 2015), tilapia (Giatsis *et al*., 2014), Atlantic cod (Forberg *et al*., 2016), Atlantic salmon (Llewellyn *et al*., 2016) and some commercially viable cyprinids (Eichmiller *et al*., 2016; Liu *et al*., 2016). Most of these fishes were studied in the rearing conditions fed basic diet or in wild conditions, which well demonstrated the influence of basic dietary input and environmental locations on the gut microbiota. However, so far very little is known about the effects of dietary YC on the composition and functional diversity of fish gut microbiota.

There is increasing evidence that prebiotics as non‐digestible food ingredients beneficially affects the physiology of host to improve growth performance, resistance against pathogenic bacteria or by stimulating the growth of beneficial bacteria (Li *et al*., 2014; Johnson *et al*., 2015; Pourabedin *et al*., 2015). Fully fermented YC is a dried product and contains yeast and various metabolites of yeast fermentation (Shen *et al*., 2009), as one of the promising feed additives candidates with many benefits in many terrestrial animals, which has been also used in aquaculture (Essa *et al*., 2011; Berto *et al*., 2016). Our results clearly indicated that YC dietary treatments for 10 weeks affected the diversity and composition of gut microbiota in grass carp and increased the relative abundance of potential beneficial bacteria species, especially in Treat3. Furthermore, YC dietary Treat3 significantly influenced the metabolic capacity of gut microbiota, exhibited good growth performances (weight gain rate and FCR) and biochemical parameters of fish (liver and serum T‐SOD, T‐AOC, GPT and GOT) compared with control diet. These results provide an explicit understanding how YC supplement in diet influences the gut microbiota diversity of grass carp and how much of YC addition is optimal to the host's food digestion and growth.

Although no significant differences were found in terms of alpha‐diversity between the control and YC treatment groups (Table 1), beta‐diversity analysis based on PCoA indicated a clear separate cluster between the control and YC Treat3 (Fig. 1), suggesting that the microbial complexity and enrichments were affected by YC diet in Treat3. This observation is in accordance with the study of Dimitroglou *et al*. (2010) who demonstrated a clear shift of intestinal microbial profiles with prebiotic MOS treatments, clustered into distinct groups in gilthead sea bream (Dimitroglou *et al*., 2010). On the contrary, Ran *et al*. (2015) found that yeast supplementation on Nile Tilapia exerted a significant influence on the alpha‐diversity of the autochthonous microbiota, but no significant difference was in beta‐diversity (Ran *et al*., 2015). This discrepancy is likely due to the intestinal microbiota variably depending on the environmental factors and the variety of host species.

In the current study, Fusobacteria, Bacteroidetes, Firmicutes and Proteobacteria constituted the most dominant phyla of bacterial communities in grass carp with different YC treatments for 10 weeks. This result is different from our previous study in gut microbiota of wild adult grass carp (Liu *et al*., 2016), which might be caused by diet composition (Bolnick *et al*., 2014; Liu *et al*., 2016) or fish age (Stephens *et al*., 2015) in addition to other environmental factors (Eichmiller *et al*., 2016). Our further metastats‐based analysis found that Actinobacteria significantly increased in Treat1, 2 and 3. It was known that Actinobacteria phylum includes *Collinsella*,* Bifidobacterium* (which contains some probiotic strains) (Tremaroli and Bäckhed, 2012) and *Actinomyces* (which was known as a cellulose‐degrading bacteria) (Ye *et al*., 2014), indicating that YC dietary treatments might be beneficial for potential favourable bacteria. Similar results have been previously reported in mice (Everard *et al*., 2011) and human (Geurts *et al*., 2014). Although fish in different YC dietary treatments shared a large size of OTUs comprising a core microbiota community at the genus level, a strong distinction existed. Some important cellulose‐degrading bacteria (Ye *et al*., 2014; Liu *et al*., 2016) including *Streptococcus*,* Leptotrichia* and *Pseudomonas* were differentially enriched in different YC treatments except Treat4. Intriguingly, some of the most abundant genera of the unique OTUs in Treat3 were also the important cellulose‐degrading candidates, such as *Clostridium* and *Methylobacterium*. It is well known that grass carp is a typical herbivorous fish species with a longer intestine, which need more cellulose‐degrading bacteria to effectively degrade the ingested food sources. This result is accordant with the previous study in ruminant that the presence of the live yeast resulted in a significant increase of cellulolytic bacterial species, indicating a beneficial effect of yeast on rumen fermentation (Mosoni *et al*., 2007).

There is increasing evidence that dietary supplementation of prebiotics on various terrestrial species beneficially affects the host to increase the number of health‐promoting bacteria such as *Lactobacillus* and *Bifidobacterium* in their intestinal tract, while decreasing potentially pathogenic bacteria (De Maesschalck *et al*., 2015; Johnson *et al*., 2015; Pourabedin *et al*., 2015). In the current study, distinct and diverse putative beneficial bacteria recognized as probiotics in aquaculture (Nayak, 2010; Lazado and Caipang, 2014; Dawood and Koshio, 2016) were identified in different YC dietary‐treated groups, especially in Treat3 (13.50%), approximately twofold higher than that in the control (Fig. 5). These microorganisms in the gastrointestinal tract could process indigestible carbohydrates and provide nutrients and vitamins to their host (Aron‐Wisnewsky and Clément, 2015; Hanning and Diaz‐Sanchez, 2015). However, the well‐known health beneficial bacteria *Lactobacillus* and *Bifidobacterium* were not enhanced after YC treatments, different from the studies on the effects of prebiotic XOS dietary in chinken reported by Pourabedin *et al*. (2015) and De Maesschalck *et al*. (2015). This difference could explain that the effects of prebiotics may vary depending on the concentration of the prebiotic in the diet, specific host and the length of feeding period. Another important factor should be also considered that the living environment for fish is more complicated and variable than that for terrestrial animals (De Schryver and Vadstein, 2014). The possibility with the presence of other putative favourable bacteria needs to be further confirmed.

Furthermore, we also found that certain concentration of YC (Treat3 and Treat4) feeding grass carp for 10 weeks showed good growth performances including the FCR and GR. Similar results have been previously reported that yeast supplementation could also promote growth performances and immune response of human (Possemiers *et al*., 2013), chicks (Gao *et al*., 2008), pigs (Shen *et al*., 2009) and fish species (Essa *et al*., 2011; Huang *et al*., 2015; Ran *et al*., 2015; Berto *et al*., 2016). For example, dietary supplemental YC at 2.5 g kg^−1^ on chicks distinctively improved the growth performances including the average daily gain and feed conversion (Gao *et al*., 2008). Also, Berto *et al*. (2016) and Ran *et al*. (2015) reported that dietary supplementation of yeast extract and live baker's yeast improved the growth, feed utilization and blood immunological indexes of Nile tilapia (Ran *et al*., 2015; Berto *et al*., 2016). It is known that antioxidant enzymes such as superoxide dismutase (SOD) and catalase (CAT) could protect against reactive oxygen species (ROS) that induced oxidative damage. We also found that YC dietary Treat3 significantly increased (*P *<* *0.05) the liver (Fig. 7A) and serum (Fig. 7E) T‐SOD enzymatic activity and dramatically decreased liver GPT (Fig. 7C) and GOT (Fig. 7D) enzyme activities compared with the control, which suggested that the biochemical parameters of grass carp were stimulated and host antioxidant capacity was enhanced by dietary supplementation of certain concentration of YC. A study carried out by Dong and Wang (2013) suggested that red swamp cray fish fed dietary certain dose of prebiotics FOS significantly increased the activity of SOD and the expression of immune genes. To further explore their potential use in aquaculture, the effects of YC on systemic immunity and how YC activates the responses of the antioxidant system should be considered for future experiments.

In conclusion, this study describes for the first time the comprehensive, high‐throughput analyses of gut microbiota diversity and demonstrates clear beneficial effects of YC in fish species. Our results clearly showed that YC dietary treatment modulated the composition and relative abundance of gut microbiota without changing the overall microbial structure. Importantly, the fact that certain YC treatment significantly increased variety and proportion of the putative beneficial bacteria species in grass carp could be an intestinal health‐promoting attribute, which might contribute to promote the growth performance and enhance the host antioxidant defences. Thus, these results indicated that YC could be used as a potential new feed additive for healthy aquaculture.

## Experimental procedures

### Experimental diets

The YC used in the present study was provided by the Hubei Gosign Bio‐feed, Wuhan, China. The percentage of amino acid of YC was shown in Table S2. Experimental diets were prepared by a commercial feed from Haid Feed, (Guangzhou, China) (approximately of 33.66% protein, 8.99% lipid and dry matter basis) supplemented with different levels [0% as Control, 8% (Treat1), 10% (Treat2), 12% (Treat3) and 16% (Treat4)] of YC (Fig. S8). After homogenization, the mixture was pelleted using a grinder (4.5 mm diameter) with 30% water incorporation, and then dried at room temperature and stored in −20 °C.

### Fish rearing conditions

A total of 500 healthy grass carp (*Ctenopharyngodon idella*) (100 ± 5 g) were obtained from Bairong fish breeding base of Huazhong Agricultural University (HZAU). The fish were acclimated to the laboratory rearing conditions for 2 weeks. During this period, a commercial pellets diet was fed twice a day. Thereafter, fish were randomly distributed into fifteen 100‐l tanks (30 fish per tank and triplicate tanks per treatment) and connected to a water‐recirculating system kept at 26 ± 1.5 °C, pH 7 ± 0.5. All tanks were cleaned every 3 days with a 30% water renewal. Each experimental diet was fed to apparent satiety twice a day (09:00 and 16:00 hours) for 10 weeks. Any uneaten feed was collected in 1 h after feeding and dried at 70 °C.

### Sample collection and growth performances

All the experimental procedures involving fish were performed in accordance with the guidelines of National Institute of Health Guide for the Care and Use of Laboratory Animals and approved by the Research Ethics Committee of Huazhong Agricultural University.

To determine whether the YC dietary treatment promotes the growth of grass carp, at the end of the feeding trial, the total number, individual body weight and length of fish from each tank were measured. After a 24‐h fasting period, all fish were euthanized with an overdose of anaesthetic and then sacrificed to obtain samples. The SR, GR, FCR and CF were determined and calculated according to the method as described by Dawood *et al*., (2016). Gain rate was calculated as [weight gain (g)/start weight (g)]. Feed conversion ratio was calculated as [feed eaten (g)/weight gained (g)]. Condition factor was calculated as 100 × [live body weight (g)/length (cm)].

### Liver and serum biochemical parameters

Blood was collected from the caudal vein using a 1 ml syringe. Serum samples were obtained by centrifugation at 3000*× g* for 15 min at 4 °C to collect serum and maintained at −80 °C for further analysis. Liver was aseptically collected and immediately homogenized with 10 volumes of cold PBS buffer (pH 6.8). The homogenate was centrifuged at 12 000× *g* for 20 min at 4 °C and the supernatant was divided into four Eppendorf tubes and then stored at −80 °C until analysis. To explore whether different concentrations of YC dietary treatments influence the biochemical parameters of liver and serum, the activities of total superoxide dismutase (T‐SOD), total antioxidant capacity (T‐AOC), glutamate pyruvate transaminase (GPT) and glutamate oxalate transaminase (GOT) enzymes were determined using assay kits (Nanjing Jiancheng Bioengineering Institute, Nanjing, China) according to the manufacturer's protocols.

### DNA extraction for gut content

To avoid transient bacteria, the whole intestinal tract of individual fish was dissected with sterile instruments and washed in 70% ethanol and sterile water. The gut content from the midgut to hindgut region was squeezed out and mixed thoroughly, then collected into sterilized tubes and immediately stored at liquid nitrogen. Bacterial genomic DNA extraction was carried out from 100 mg of each sample with a QIAamp DNA Stool Mini Kit (Qiagen, Valencia, CA, USA) following the manufacturer's protocol. The concentration of each DNA sample was quantified using NanoDrop ND‐2000 spectrophotometer (Thermo Scientific, Wilmington, DE, USA) and diluted to the same concentration for subsequent PCR amplification.

### Amplification, sequencing, assemblage and determination of taxonomic units of fragments

To examine the microbial communities of each sample, a total of 43 libraries, corresponding to nine control, nine Treat1, eight Treat2, eight Treat3 and nine Treat4 were constructed and sequenced using the Illumina MiSeq platform (Illumina, San Diego, CA, USA) with 2 × 250 bp kits following the manufacturer's instructions. The bacterial V3‐V4 hypervariable region of the 16S ribosomal gene was amplified using the following primers: 319F: 5′‐ACTCCTACGGGAGGCAGCAG‐3′) and 806R: 5′‐GGACTACHVGGGTWTCTAAT‐3′ according to the previously described methods (Fadrosh *et al*., 2014). All control and YC‐treated samples were included in the same sequencing run. All sequences have been deposited in the NCBI's Sequence Read Archive (SRA accession number to be provided upon acceptance).

FLASH (Magoč and Salzberg, 2011) was used to merge the overlapping paired‐end reads into single longer reads covering the full 16S rRNA V3‐V4. UCHIME (Edgar *et al*., 2011) was used to identify possible chimeric sequences and the unique sequences were aligned using the SILVA database (Pruesse *et al*., 2007). The filtered and trimmed high‐quality reads were further processed to analyse the microbial community composition. Briefly, the UCLUST (Edgar, 2010) with 97% similar was used to cluster of nearly identified reads as an OTU, then representative sequences of each OTU were aligned to the most recently available version of the bacterial database Green genes using PyNAST (Caporaso *et al*., 2010). Taxonomic assignment was achieved using RDP Classifier (Wang *et al*., 2007), clustering the sequences at 97% similarity with a confidence threshold of 0.80. The relative abundance of OTUs and microbial composition in different taxonomic levels was calculated and analysed for each group.

### Parameter calculation and statistical analysis on microbiota diversity

Alpha‐diversity metrics including observed OTUs, rarefaction curve, ACE (Eckburg *et al*., 2006), Chao1 (Chao, 1984) and Shannon's diversity (Shannon, 1948) index were calculated on rarefied OTU tables with Mothur version v.1.30 (Schloss *et al*., 2009). The overall microbiome dissimilarities among all gut content samples were ordinated using the weighted and unweighted UniFrac distance metrics (Lozupone and Knight, 2005) and visualized with principal coordinates analysis (PCoA) by the R package phyloseq. Discriminatory analysis of the gut microbiome features among control and YC‐treated groups at various taxonomic ranks was performed with LEfSe (http://huttenhower.sph.harvard.edu/galaxy) (Segata *et al*., 2011) with alpha value < 0.05 for the factorial Kruskal–Wallis test among classes and threshold on the logarithmic LDA score for discriminative features > 2. To predict the differences of metagenome functional profile of the gut microbiota between control and YC‐treated groups, PICRUSt (Phylogenetic Investigation of Communities by Reconstruction of Unobserved States) (Langille *et al*., 2013) was applied using GreenGenes database. We then binned the identified KOs into functional categories under a given subsystem hierarchy at level II and level III derived from KEGG (Kyoto encyclopedia of genes and genomes) modules (Ogata *et al*., 1999). The output file was further analysed using the software package STAMP (Statistical Analysis of Metagenomic Profiles) (Parks *et al*., 2014). STAMP implements the Welch's *t*‐test for comparing profiles organized into two groups and the ANOVA with post hoc tests (Tukey–Kramer) for comparing three or more groups of profiles. Statistical comparison of two groups was performed using a Student's *t*‐test. Data sets that involved more than two groups were assessed by one‐way analysis of variance (ANOVA) with Tukey's multiple comparison followed by Newman–Keuls *post hoc* tests. All data are shown as means ± SEM unless otherwise indicated. Values were considered statistically significant when *P *<* *0.05. The statistical analyses were performed with SPSS 19.0 (SPSS, Chicago, IL, USA).

## Conflict of interest

The authors declare no conflict of interest.

## Supporting information


**Fig. S1.** Rarefaction analyses of gut samples with different YC dietary treatments.
**Fig. S2.** The relative abundance of gut microbiota in individual grass carp at phylum level.
**Fig. S3.** Relative abundance of four taxa at phylum level significantly associated with YC treatments.
**Fig. S4.** Relative abundance of six taxa at genus level significantly associated with YC treatments.
**Fig. S5.** Differences in the gut microbiota of grass carp between control and YC dietary Treat3.
**Fig. S6.** Venn diagrams showing compartmental core microbiota OTU distributions in grass carp.
**Fig. S7.** Mean proportion and their differences in predicted functional metagenomes of the gut microbiota among YC dietary treatments.
**Fig. S8.** Schematic representation of experimental design.
**Table S1.** Detailed fish gut sample information.
**Table S2.** The percentage of amino acid in yeast cultures.
