# Supplementary material for: Yeast culture dietary supplementation modulates gut microbiota, growth and biochemical parameters of grass carp
Source: Microb Biotechnol. 2018 Mar 26;11(3):551–65. doi: 10.1111/1751-7915.13261 (PMC5902330; doi:10.1111/1751-7915.13261)
Supplement: Supplementary file 1 — Fig. S1. Rarefaction analyses of gut samples with different YC dietary treatments. Fig. S2. The relative abundance of gut microbiota in individual grass carp at phylum level. Fig. S3. Relative abundance of four taxa at phylum level significantly associated with YC treatments. Fig. S4. Relative abundance of six taxa at genus level significantly associated with YC treatments. Fig. S5. Differences in the gut microbiota of grass carp between control and YC dietary Treat3. Fig. S6. Venn diagrams showing compartmental core microbiota OTU distributions in grass carp. Fig. S7. Mean proportion and their differences in predicted functional metagenomes of the gut microbiota among YC dietary treatments. Fig. S8. Schematic representation of experimental design. Table S1. Detailed fish gut sample information. Table S2. The percentage of amino acid in yeast cultures. [file MBT2-11-551-s001.docx]

**Supporting Information**

**Yeast Culture Dietary Supplementation Modulate** **Gut Microbiota, Growth and Biochemical Parameters of Grass Carp**

Liu Han^1^, Li Juntao^1^, Guo Xianwu^3^, Liang Yunxiang^1^, Wang Weimin^1,2*^

^1^College of Fisheries, Key Lab of Freshwater Animal Breeding, Ministry of Agriculture, Key Lab of Agricultural Animal Genetics, Breeding and Reproduction of Ministry of Education, Huazhong Agricultural University, Wuhan 430070, China

^2^Collaborative Innovation Center for Efficient and Health Production of Fisheries in Hunan Province, Changde 41500, China

^3^Laboratorio de Biotecnología Genómica, Centro de Biotecnología Genómica, Instituto Politécnico Nacional, Boulevard del Maestro esquina Elías Piña, Colonia Narciso Mendoza, Ciudad Reynosa, 88710, Tamaulipas, Mexico

^*^Corresponding author:

Weimin Wang, PhD, Professor

E-mail address: wangwm@mail.hzau.edu.cn

Tel: +86-27-8728 4292; Fax: +86-27-8728 4292

^*^Current address: College of Fisheries, Huazhong Agricultural University, Wuhan 430070, China

**Figure S1**


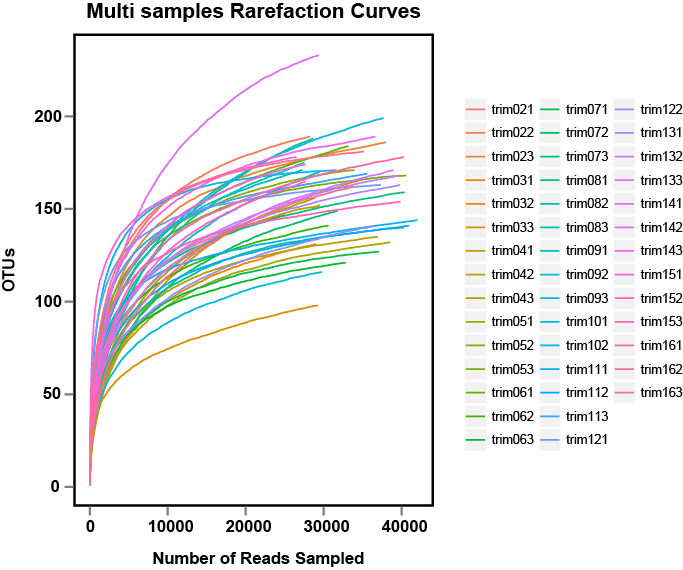


Fig. S1 Rarefaction analyses of gut samples with different YC dietary treatments. Rarefaction curves analysis showing the number of detected OTUs per sample based on sequence number for 43 specimens. Sequences are clustered with similarity cut-off of 97%.

**Figure S2**


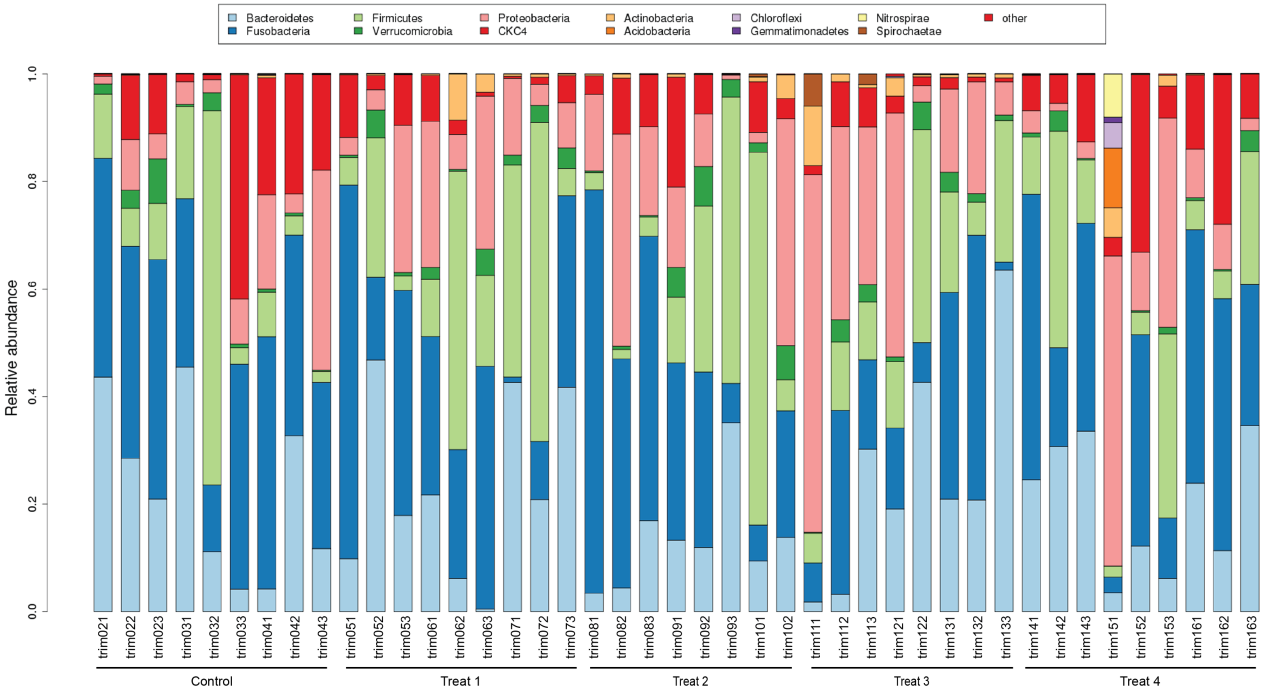


Fig. S2 The relative abundance of gut microbiota in individual grass carp at phylum level. Treat1, 2, 3, 4 represent the samples with different YC dietary treatments.

**Figure S3**


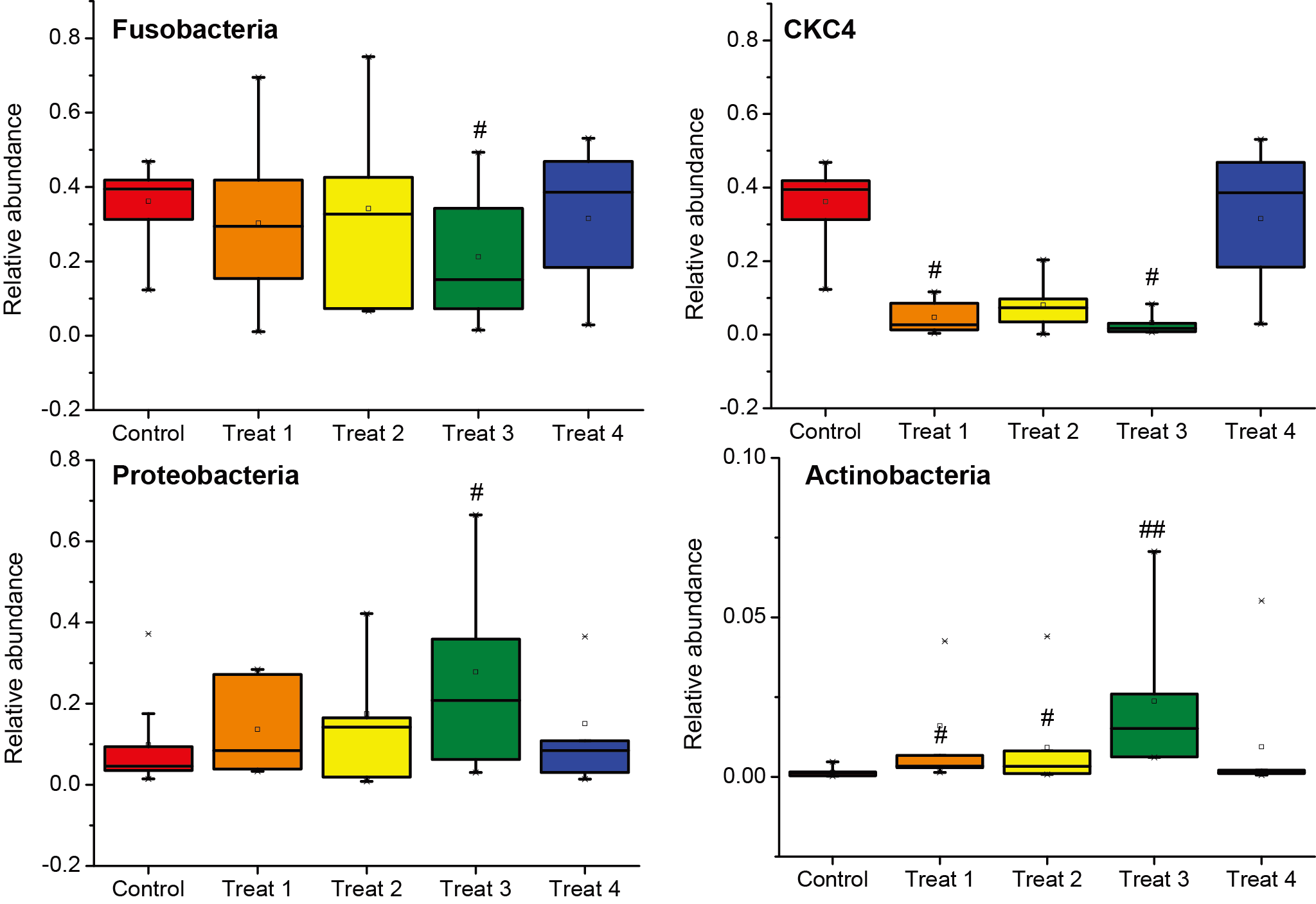


Fig. S3 Relative abundance of four taxa at phylum level significantly associated with YC treatments. ^#^*P* < 0.05, ^##^*P* < 0.01

**Figure S4**


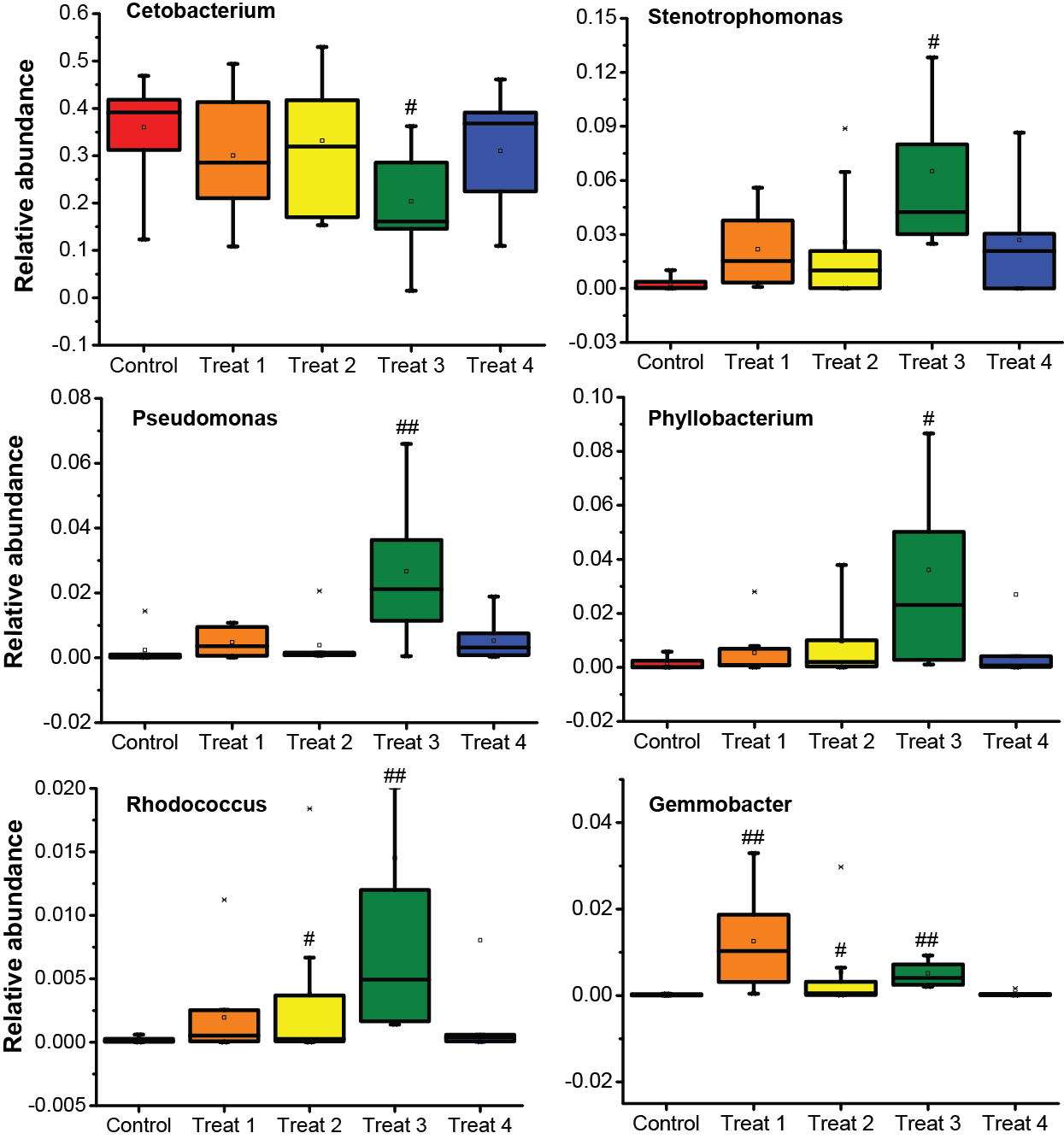


Fig. S4 Relative abundance of six taxa at genus level significantly associated with YC treatments.

^#^*P* < 0.05, ^##^*P* < 0.01

**Figure S5**


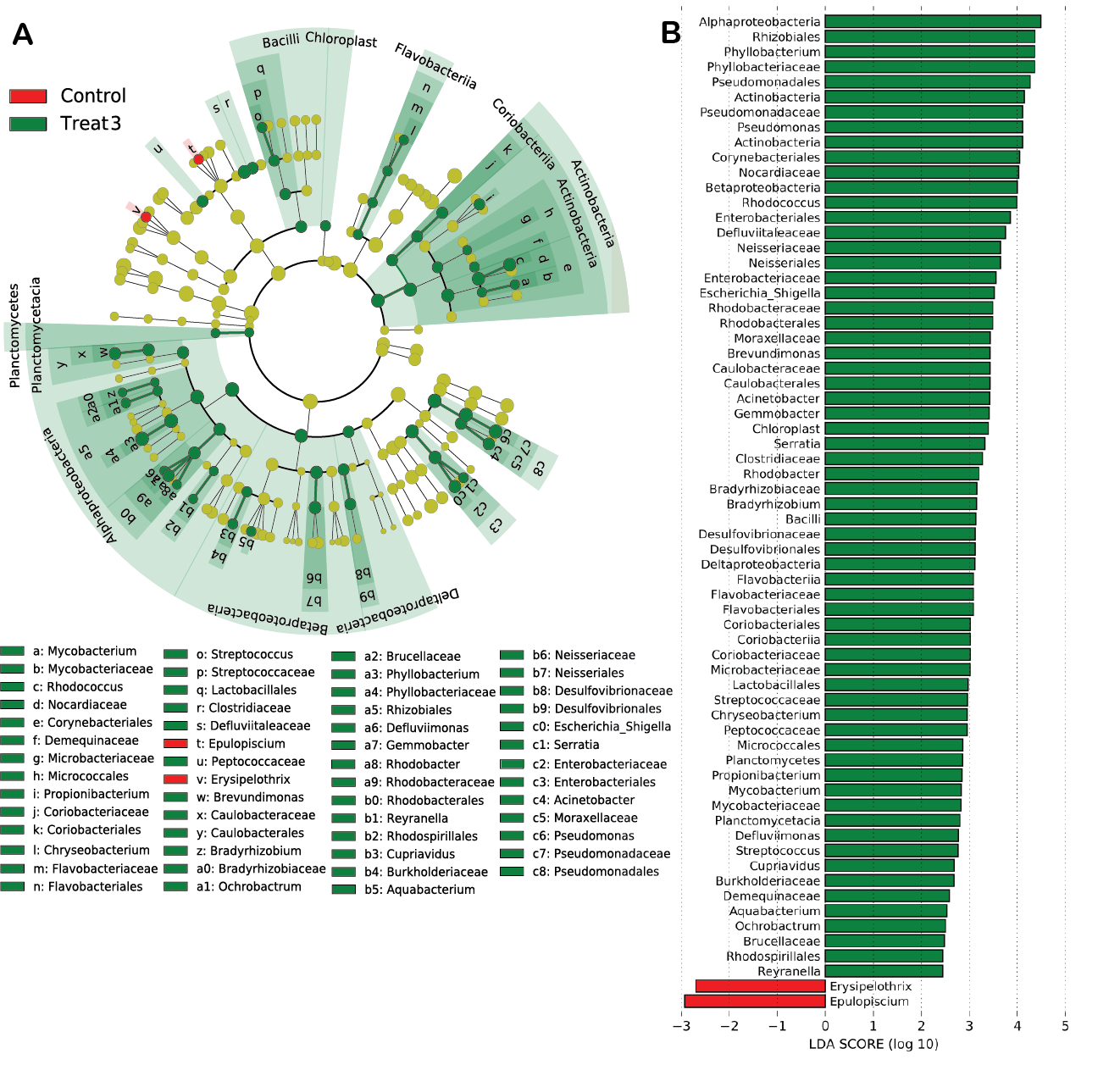


Fig. S5 Differences in the gut microbiota of grass carp between control and YC dietary Treat3.

**Figure S6**


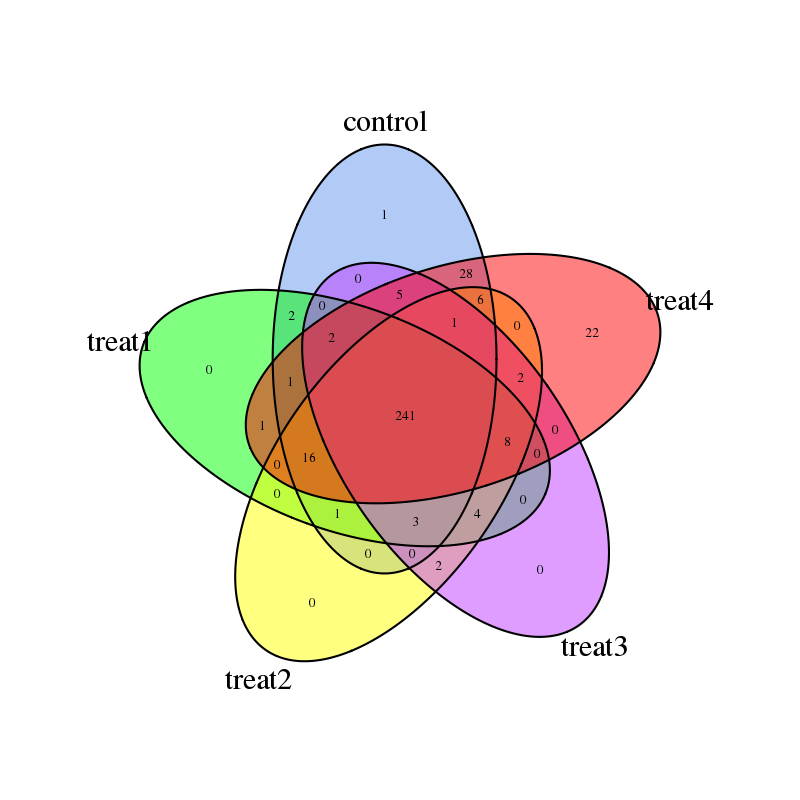


Fig. S6 Venn diagrams showing compartmental core microbiota OTU distributions in grass carp.

**Figure S7**


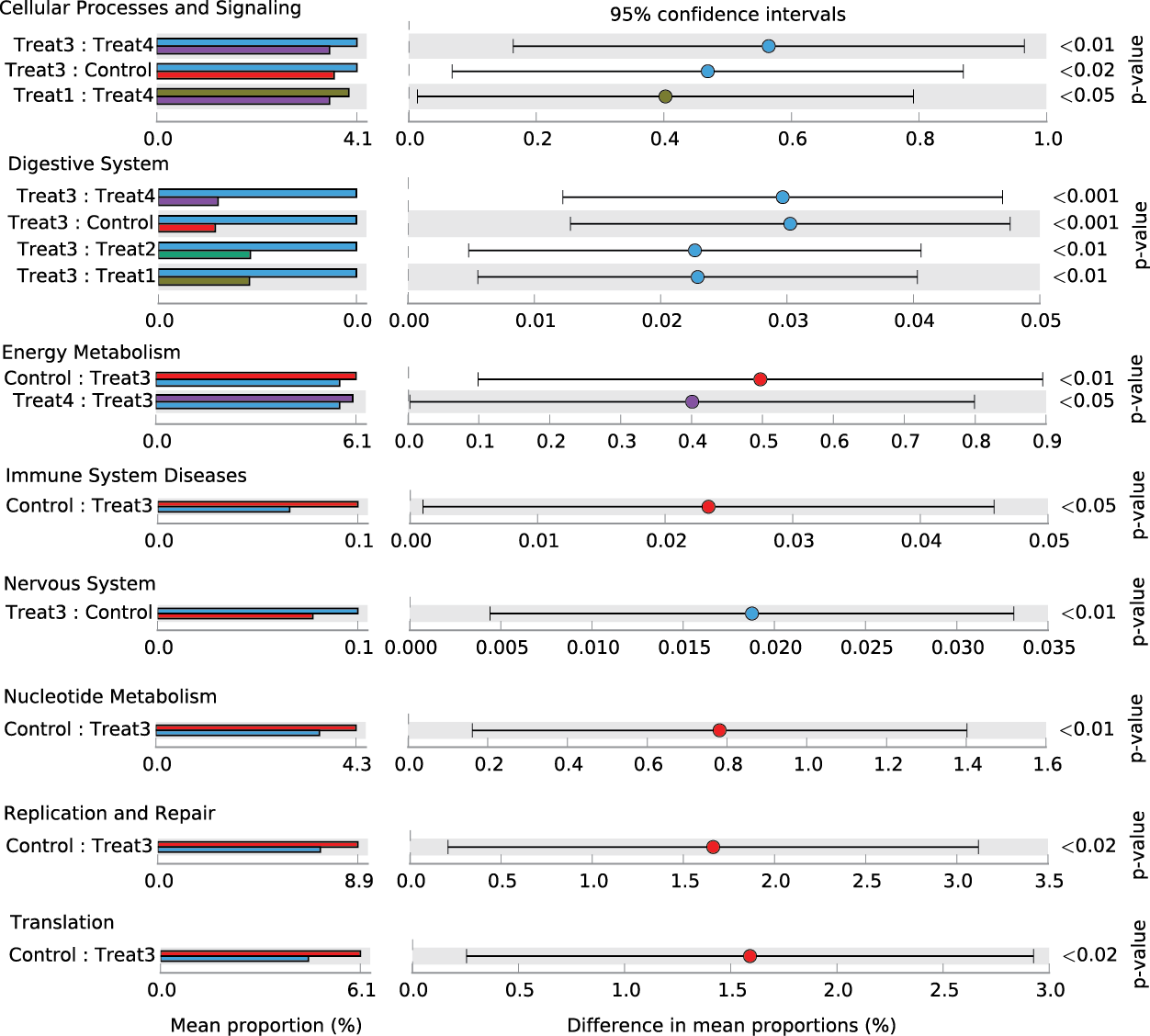


Fig. S7 Mean proportion and their differences in predicted functional metagenomes of the gut microbiota among YC dietary treatments. Control in red, Treat1 in brown, Treat2 in green, Treat3 in blue and Treat4 in purple.

**Figure S8**


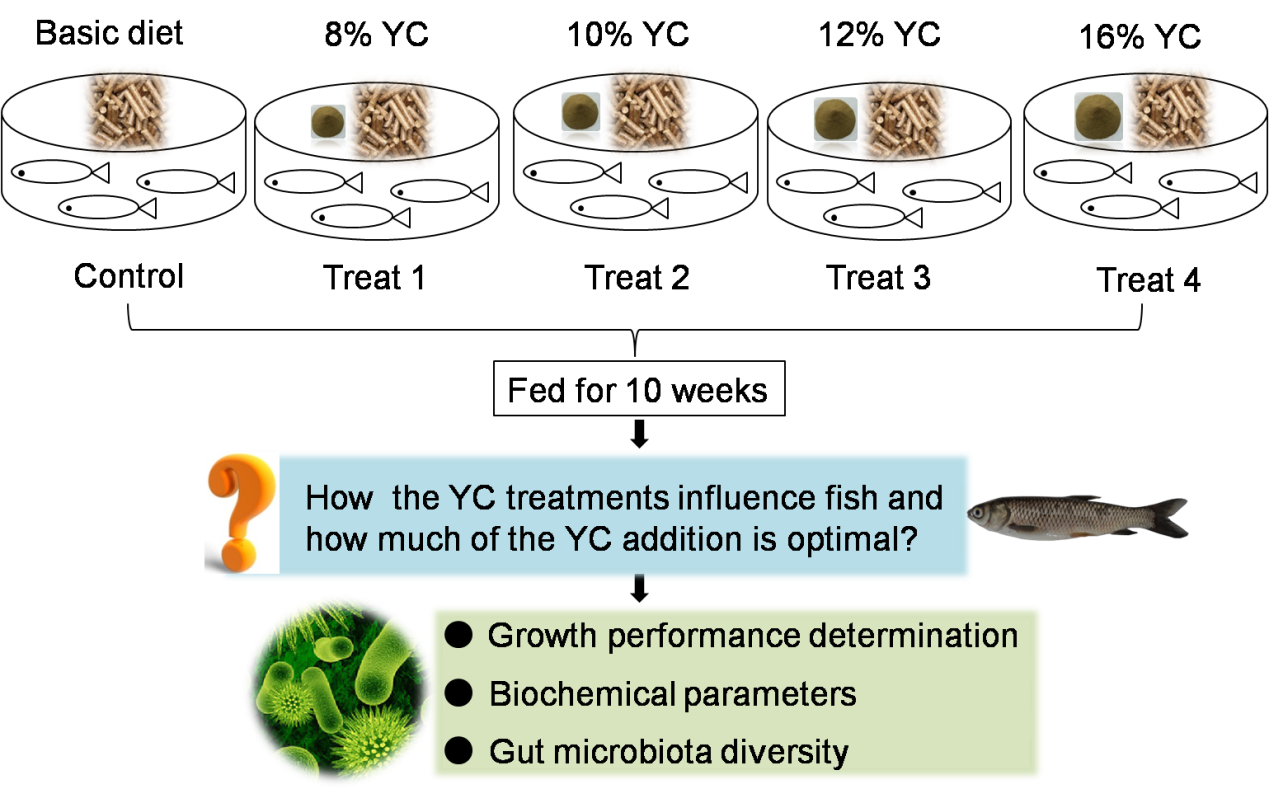


Fig. S8 Schematic representation of experimental design.

**Supporting Information (Tables)**

**Table S1 Detailed fish gut sample information**

| **Groups** | **Sample ID** | **Raw Tags** | **Clean Tags** | **Average**  **Length (bp)** | **GC**  **(%)** | **Q20**  **(%)** | **Q30**  **(%)** | **Effective (%)** |
| --- | --- | --- | --- | --- | --- | --- | --- | --- |
| Control | 021 | 30518 | 26203 | 436 | 48.08 | 99.37 | 97.54 | 85.86 |
| Control | 022 | 31257 | 28917 | 438 | 49.3 | 99.39 | 97.6 | 92.51 |
| Control | 023 | 42899 | 39010 | 436 | 49.47 | 99.31 | 97.3 | 90.93 |
| Control | 031 | 32157 | 29438 | 437 | 48.23 | 99.27 | 97.17 | 91.54 |
| Control | 032 | 32708 | 30093 | 437 | 49.81 | 99.19 | 96.87 | 92.01 |
| Control | 033 | 37677 | 35315 | 440 | 50.52 | 99.29 | 97.18 | 93.73 |
| Control | 041 | 31674 | 30139 | 438 | 50.66 | 99.32 | 97.32 | 95.15 |
| Control | 042 | 40998 | 37554 | 439 | 48.63 | 99.38 | 97.53 | 91.6 |
| Control | 043 | 39988 | 38841 | 442 | 50.41 | 99.32 | 97.34 | 97.13 |
| Treat 1 | 051 | 32515 | 29267 | 433 | 49.03 | 99.44 | 97.79 | 90.01 |
| Treat 1 | 052 | 38639 | 35395 | 439 | 48.75 | 99.35 | 97.43 | 91.6 |
| Treat 1 | 053 | 43401 | 41011 | 439 | 49.96 | 99.32 | 97.33 | 94.49 |
| Treat 1 | 061 | 35051 | 33475 | 440 | 50.21 | 99.22 | 96.89 | 95.5 |
| Treat 1 | 062 | 31865 | 31122 | 432 | 51.7 | 99.36 | 97.49 | 97.67 |
| Treat 1 | 063 | 34981 | 33348 | 433 | 50.95 | 99.41 | 97.67 | 95.33 |
| Treat 1 | 071 | 37603 | 37430 | 438 | 49.38 | 99.29 | 97.3 | 99.54 |
| Treat 1 | 072 | 33326 | 32293 | 435 | 49.86 | 99.01 | 96.51 | 96.9 |
| Treat 1 | 073 | 42926 | 40966 | 439 | 48.32 | 99.33 | 97.28 | 95.43 |
| Treat 2 | 081 | 33207 | 29044 | 433 | 49.55 | 99.39 | 97.6 | 87.46 |
| Treat 2 | 082 | 42596 | 40562 | 439 | 50.96 | 99.33 | 97.32 | 95.22 |
| Treat 2 | 083 | 30351 | 27831 | 437 | 49.34 | 99.34 | 97.43 | 91.7 |
| Treat 2 | 091 | 32302 | 28233 | 439 | 50.49 | 99.26 | 97.12 | 87.4 |
| Treat 2 | 092 | 31683 | 29844 | 437 | 50.14 | 99.33 | 97.32 | 94.2 |
| Treat 2 | 093 | 30816 | 30028 | 437 | 49.15 | 99.3 | 97.24 | 97.44 |
| Treat 2 | 101 | 39541 | 38611 | 430 | 51.35 | 99.44 | 97.78 | 97.65 |
| Treat 2 | 102 | 33783 | 32698 | 439 | 51.2 | 99.26 | 97.1 | 96.79 |
| Treat 3 | 111 | 43481 | 43032 | 439 | 53.21 | 99.16 | 96.77 | 98.97 |
| Treat 3 | 112 | 43039 | 41527 | 438 | 51.24 | 98.9 | 96.18 | 96.49 |
| Treat 3 | 113 | 36830 | 36085 | 442 | 50.1 | 99.31 | 97.26 | 97.98 |
| Treat 3 | 121 | 40312 | 39556 | 441 | 51.06 | 99.28 | 97.07 | 98.12 |
| Treat 3 | 122 | 37094 | 36784 | 438 | 49.1 | 99.39 | 97.57 | 99.16 |
| Treat 3 | 131 | 41910 | 40081 | 437 | 49.62 | 99.31 | 97.3 | 95.64 |
| Treat 3 | 132 | 43003 | 40010 | 436 | 49.36 | 99.32 | 97.35 | 93.04 |
| Treat 3 | 133 | 41025 | 39570 | 442 | 48.15 | 99.05 | 96.67 | 96.45 |
| Treat 4 | 141 | 32443 | 29866 | 435 | 48.69 | 99.4 | 97.64 | 92.06 |
| Treat 4 | 142 | 42488 | 38585 | 436 | 49.37 | 99.36 | 97.48 | 90.81 |
| Treat 4 | 143 | 30189 | 28132 | 438 | 48.61 | 99.29 | 97.2 | 93.19 |
| Treat 4 | 151 | 35239 | 34908 | 438 | 54.44 | 99.25 | 97.01 | 99.06 |
| Treat 4 | 152 | 30418 | 28032 | 440 | 50.14 | 99.31 | 97.25 | 92.16 |
| Treat 4 | 153 | 41144 | 40516 | 434 | 51.76 | 99.26 | 97.15 | 98.47 |
| Treat 4 | 161 | 44625 | 40822 | 437 | 49.03 | 99.4 | 97.6 | 91.48 |
| Treat 4 | 162 | 35754 | 33993 | 438 | 49.99 | 99.37 | 97.49 | 95.07 |
| Treat 4 | 163 | 42736 | 37119 | 438 | 48.93 | 99.32 | 97.34 | 86.86 |

**Table S2 The Percentage of Amino Acid in Yeast Cultures**

| **Items** | **Measured Value** |
| --- | --- |
| Cystine (%) | 0.25 |
| Tryptophan (%) | 0.17 |
| Aspartic (%) | 1.66 |
| Threonine (%) | 0.68 |
| Serine (%) | 0.82 |
| Glutamic (%) | 3.77 |
| Glycine (%) | 0.80 |
| Alanine (%) | 1.35 |
| Valine (%) | 1.10 |
| Methionine (%) | 0.37 |
| Isoleucine (%) | 0.54 |
| Leucine (%) | 2.05 |
| Tyrosine (%) | 0.54 |
| Phenylalanine (%) | 0.94 |
| Lysine (%) | 0.88 |
| Histidine (%) | 0.63 |
| Arginine (%) | 0.67 |
| Proline (%) | 1.20 |
